# Urogenital Hiatus Closure System: A Framework for Understanding how Muscle, Motor Control, and Fascial Connections Interact in Normal and Failed Closure

**DOI:** 10.1007/s00192-026-06641-4

**Published:** 2026-04-15

**Authors:** John O. L. DeLancey, James A. Ashton-Miller, Jennifer LaCross, Payton Schmidt, Mariana Masteling, Fernanda Pipitone, Christopher X. Hong, Luyun Chen

**Affiliations:** 1 Department of Obstetrics and Gynecology, University of Michigan, 1500 E. Medical Center Dr., Ann Arbor, MI 48109 USA; 2 Department of Mechanical Engineering, University of Michigan, Ann Arbor, MI USA; 3 Hospital das Clinicas FMUSP, University of Sao Paulo, São Paulo, Brazil

**Keywords:** Pelvic organ prolapse, Urogenital hiatus, Levator ani muscles, Perineal membrane, Perineal body, Neuromotor control, Review, Pathophysiology

## Abstract

Failure of the urogenital hiatus to remain closed in women is a major contributor to prolapse operation failure and development of prolapse after childbirth. This article presents a conceptual framework, the Urogenital Hiatus Closure System (UHCS), that explains how anatomic and neuromuscular elements interact to maintain, or fail to maintain, hiatal closure. Clinical observations demonstrate that no single structure reliably predicts hiatus size; instead, hiatal behavior results from the integrated functional components. The UHCS has three primary elements: the levator ani muscle, neuromotor control, and Level III connective tissues of the perineal complex—each of which can be injured, partially compensated, or overloaded. These structures form a neuromechanically integrated unit in which the medial levator ani, the perineal membrane and its central connection, and the afferent–efferent control loops work to provide resting tone, active contraction, resistance to dilation, and spatial alignment. A principle of the model is redundancy: failure in one component may not enlarge the hiatus, but combinations of failures exceed compensatory capacity and result in failure. It links Level III mechanics to Level I–II support by demonstrating how an open hiatus increases forces on apical and paravaginal tissues, driving a feed-forward cycle of prolapse dilation and muscle elongation. Conceptually, the UHCS is an interacting triad influenced by functional modifiers—loading conditions, prolapse effects, muscle length, motor activation, and connective-tissue properties—that determine hiatus size. This systems-based approach can guide classification of failure patterns to inform biomechanical, anatomical, and therapeutic research.

## Introduction

It has been hypothesized for over a century that an enlarged urogenital hiatus plays an important role in causing pelvic organ prolapse [1, 2]. Clinical measurements supporting this hypothesis demonstrate that not only is pelvic organ prolapse associated with increased urogenital hiatus size, but also that it was larger after failed operations than after successful surgery [3]. Since then, in a growing body of research on measurements of the levator and urogenital hiatuses in pregnancy, postpartum, and with pelvic floor disorders, failure of the urogenital hiatus to remain closed (or resist enlargement) has emerged as an important factor in understanding pelvic floor disorders [4].In this article, we will focus on the urogenital hiatus because it can be easily assessed during any pelvic examination and due to its structural complexity. Levator hiatus measures are strongly correlated with urogenital hiatus measures.

**Fig. 1:**
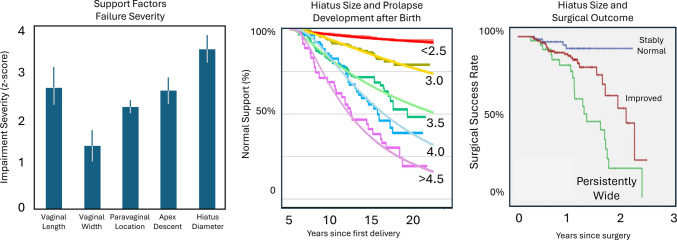
Hiatus size, severity, and outcomes. Data showing that increased hiatal diameter is the most severe support system failure site [5], associated with prolapse development after birth [6], and associated with operative failure [7]

Compared to loss of apical support, paravaginal descent, and vaginal fascial widening/lengthening that represent Level I and II support, hiatus enlargement in Level III is the most severe of all these sites (Fig. 1) [5]. As we will explain later, hiatal closure and the Level I and II connective tissue attachments are interrelated. Failure of Level III increases the loads that must be carried by connective tissues that, over time, can lead to their failure. The development of prolapse is greatly increased in women who have an enlarged hiatus five years after delivery [6], and women with a persistently enlarged hiatus after surgery are more likely to have their prolapse recur than those with improved or stably normal hiatuses [7]. Therefore, both obstetricians and gynecologists can benefit from a better understanding of failure mechanisms.

Our earlier work proposed a pelvic floor conceptual model for the overall pelvic floor [8], with a subsequent publication of data based on the model [9]. These data reconfirmed the importance of hiatal closure and evaluated the interplay of pelvic floor descent (levator bowl volume, levator plate shape) and levator muscle length with hiatus size. However, they did not address the anatomical structures involved in urogenital hiatus closure. Recent anatomical studies have clarified the interconnections and interactions among several structures surrounding the urogenital hiatus—referred to as the “perineal complex” [10]. These structures have a unique arrangement and represent diverse tissue types and a sophisticated control system. As such, a specific closure system framework is needed to help guide obstetrical, gynecological, and biomechanical research into the different failure patterns responsible for an enlarged hiatus.

### Clinical Observations About Hiatal Closure

This article describes a framework for understanding the Urogenital Hiatus Closure System (UHCS) in Level III, which considers the structures and interactions necessary to understand hiatal closure. Development of such a framework must be guided by the criterion for something to be considered "true”—that is, it cannot be proven false by empirical observation. We recently published photographic evidence of six empirical observations about hiatal status in women with which a UHCS model must agree [11]. The essentials of these observations are summarized in Table 1. Observations 1 and 2 demonstrate that failure in any single element does not always result in hiatal enlargement, requiring the Urogenital Hiatus Closure System (UHCS) model. This suggests that hiatal enlargement should be evaluated with a multi-hit paradigm that assesses the status of all aspects of the system and considers their interactions. Observations 3 and 4 show that measurements of hiatal size differ depending on the dilating force of a prolapse and the strength of a Valsalva maneuver. Observation 5 implies that assessment of hiatal closure must consider the ability of the levator ani muscle to volitionally contract and, ideally, the automatic physiological adjustments that occur in the muscle with varying loads. Observation 6 suggests that changes in the hiatus with advancing age are different if a pre-existing injury is present.

**Table 1:** Hiatus observations with which a theory must be consistent^a^

Observation	Evidence
1: Absent perineal body paradox	Tight hiatal closure can occur despite total absence of the perineal body in women with 4th degree laceration
2: Levator ani defect discrepancy	The hiatus can be perfectly normal with bilateral avulsion yet greatly enlarged with intact levators
3: Straining-resting discordance	Two women with identical enlarged hiatuses during straining can have resting hiatuses that are four-fold different in size
4: Hiatus-straining inconsistency	Hiatus size varies with straining effort and prolapse protrusion in a single individual
5: Muscle action difference	Some women with an enlarged hiatus can close it with a pelvic muscle contraction while others cannot
6: Hiatus alteration with aging	Normal hiatuses stay normal but abnormal ones enlarge with advancing age

^a^see DeLancey et al. [11] for corresponding photographic documentation

Given the importance of hiatal enlargement, we need to understand the structural failures—and combinations of failures—that are responsible for failed closure so that treatment can be targeted to the specific failure present in a patient. It is also probable that novel treatments need to be developed for failures that do not currently have a treatment. These insights might also help in developing novel prevention strategies and in the objective assessment of these strategies’ success.

### Overview of System Elements

**Fig. 2:**
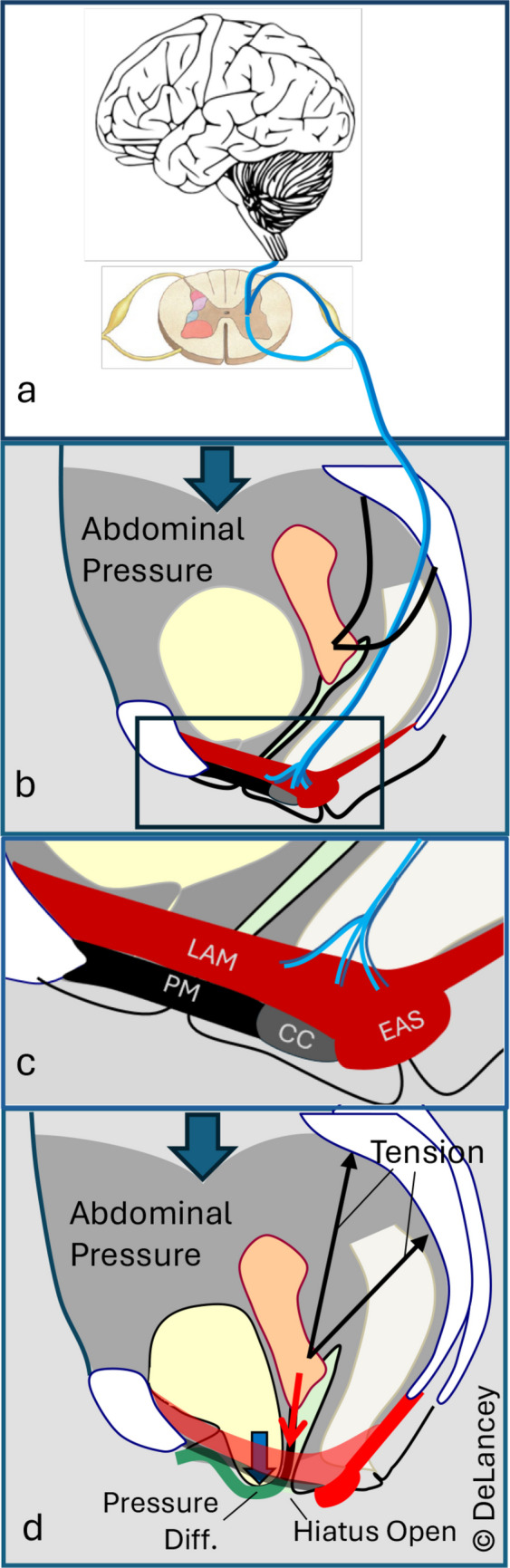
The urogenital hiatus control system in pelvic support (**a**, **b**, **c**) Schematic of the perineal complex: levator ani muscles (LAM), perineal membrane (PM), central connection (CC), and afferent (dark blue) and efferent (light blue) aspects of neural control. EAS, external anal sphincter; increased abdominal pressure (down arrow) **d**) Effect of hiatal opening on ligament tensions due to pressure differential on the anterior vaginal wall by traction on the uterus

An overall schematic of the role of the UHCS in pelvic organ support is shown in Fig. 2. This is a multifaceted arrangement consisting of connective tissue, striated muscle, and a neural control system. This latter component includes both afferent and efferent nerves (e.g., gamma motor neurons)—reflexive neurological mechanisms occurring at the level of the spinal cord and brain (i.e., motor cortex, somatosensory cortex). Pelvic floor structures include the levator ani muscles, nerve to the levator ani, perineal membranes, and the varied connective tissues that unite them—especially the central connections in the region of the perineal body [10]. (Note that the term “perineal body” is a regional term such as “knee”—not a name for a specific structure, like patella or meniscus.) The central connection is the region where the two sides of the puboperineal part of the pubococcygeal muscle and the superficial transverse perineal muscle unite to form an area where the bulbospongiosus muscles inserts [12].

The interaction between Level I and II and an open hiatus is shown in Fig. 2d. A key element of pelvic floor function is the way in which hiatal opening and ligament tension interact. Normally, when the hiatus is closed (Fig. 2b), increases in abdominal pressure result in roughly equal anterior and posterior compartment pressures [13] that cancel each other out. However, when the hiatus is open and the vaginal wall is exposed to the difference between high abdominal pressure and low atmospheric pressure, a force is created that places Level I and II structures under tension (Fig. 2d). Since pressure is force per unit area (e.g., lbs. per in^2^), the larger the hiatal area, the larger the force. Because the area of a circle increases by the square, doubling the radius would increase the area four-fold and quadruple the force resulting from the same pressure.

**Fig. 3:**
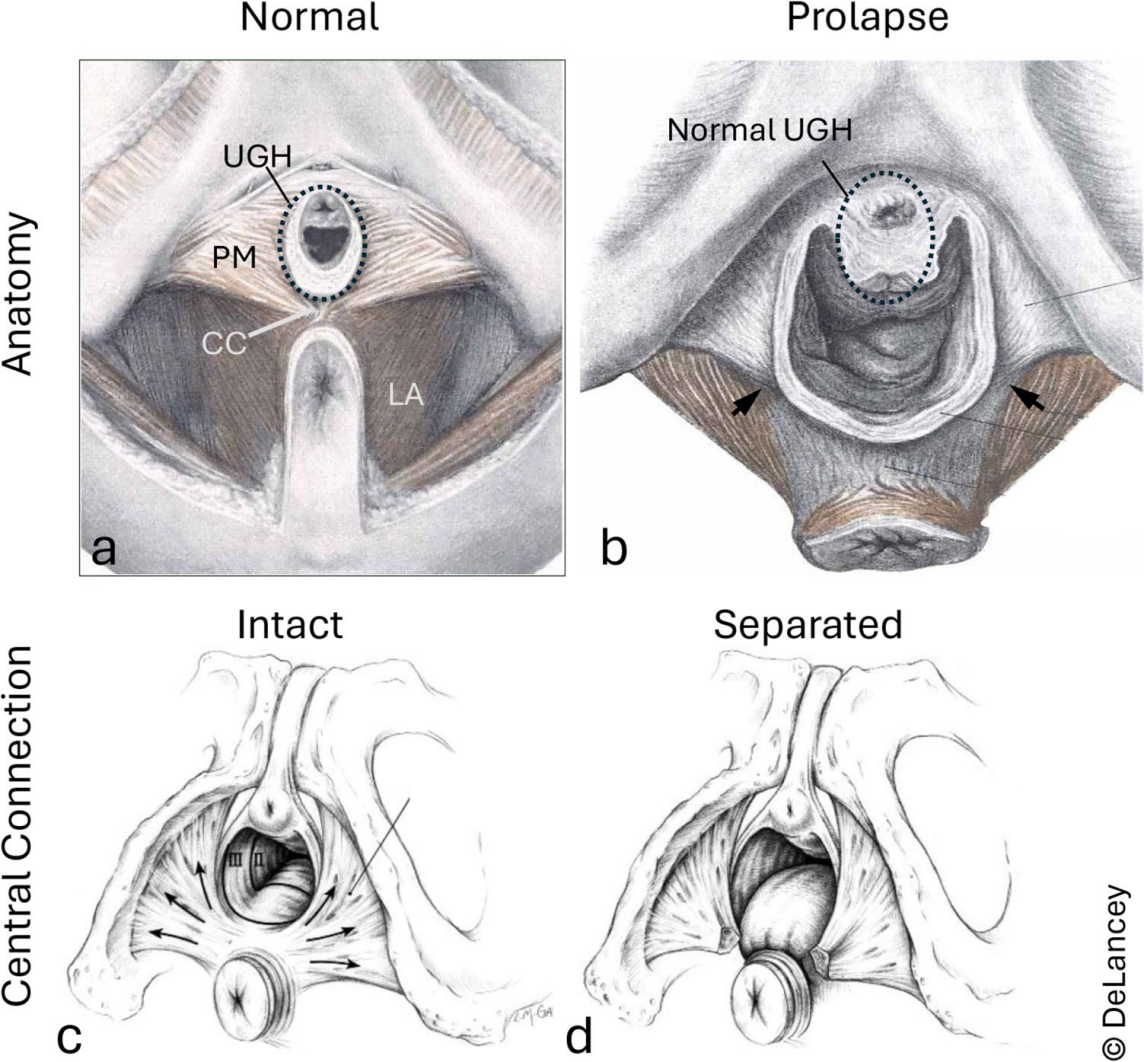
Relationships between perineal complex structures. Dissection of the perineal complex from cadavers with normal support (**a**) and prolapse (**b**). Note differences between structures surrounding the normal urogenital hiatus (UGH) and enlarged hiatus, including separated perineal membranes (PM) and diastasis of the levator ani (**LA**) muscles (arrows). The mechanical effect of central connection failure is also illustrated in (**c**) and (**d**). Panels a and b from Halban and Tandler.^1^ Panels c and d, ©DeLancey

Dissections of a normal cadaver and one with prolapse (Fig. 3) illustrate the relationships between the different elements of the perineal complex. For example, loss of the central connection between the two sides can result in diastasis of the levator ani muscles and hiatal enlargement [14]. The geometry of these connections, as well as the muscular and neuromuscular control mechanisms necessary to be consistent with Observation 5 and properties of the connective tissues, must be understood because alterations in one area may have consequences in other parts of the complex.

**Fig. 4:**
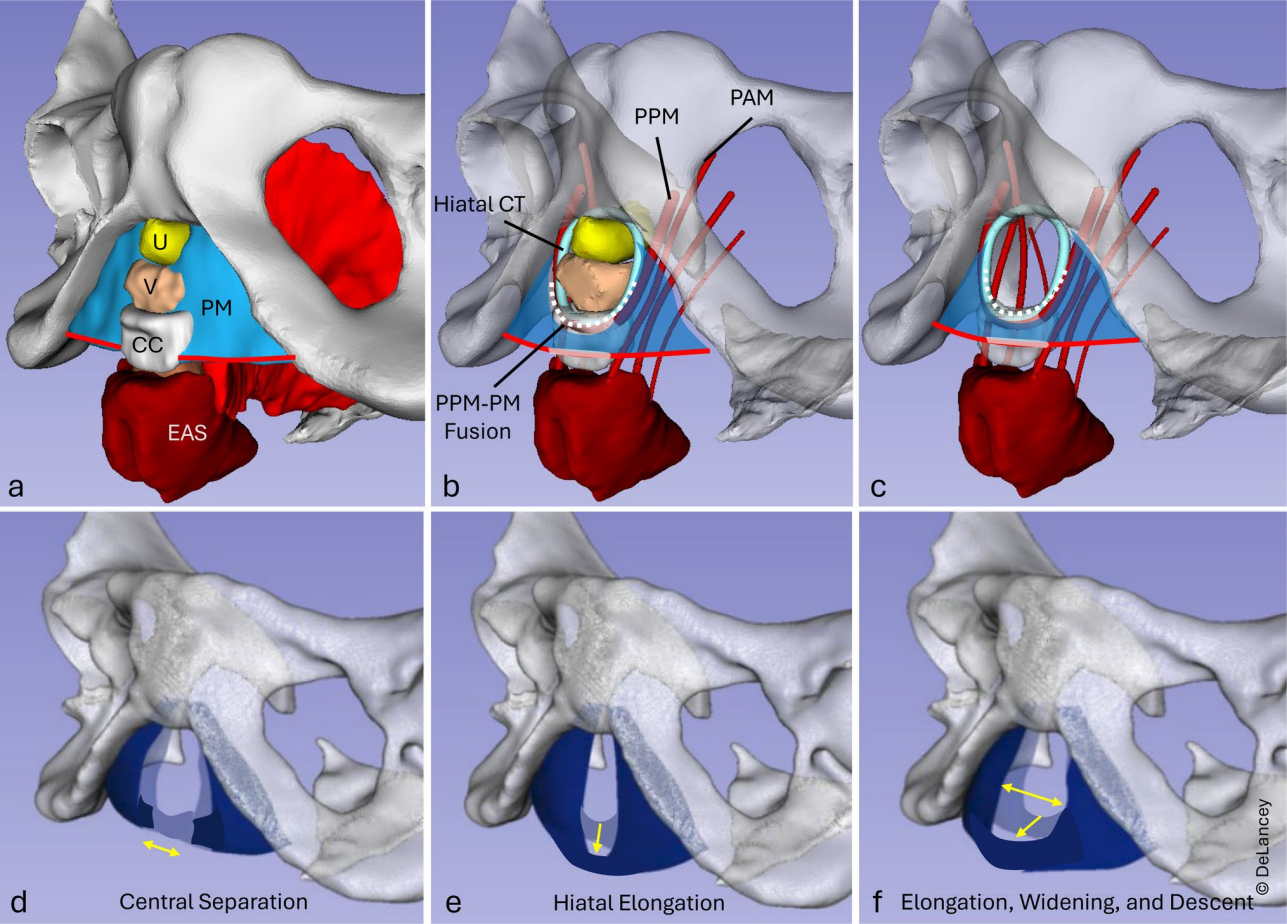
3D model of the perineal complex. Model of the perineal complex from the MRI of a nullipara with normal support (**a**-**c**). Teal ring, hiatal connective tissue (Hiatal CT); white dashed line, perineal membrane (PM) and levator ani fusion. (CC, central connection; EAS, external anal sphincter; U, urethra; V, vagina; Levator ani subdivisions: PPM, puboperineal; PAM, puboanal). Bottom row demonstrates examples of perineal membrane changes. Light blue perineal membrane, normal configuration; dark blue, distortion

**Fig. 5:**
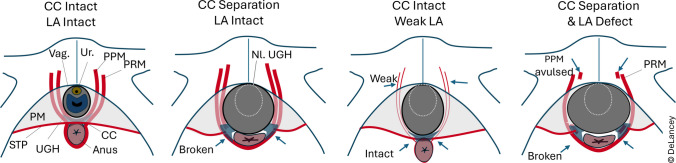
Examples of hiatal failure sites and combinations. Diagrammatic representation of key hiatal closure elements with examples of different hypothesized failures and how they would affect hiatus size and shape. Levator weakness shown in the third example can come from several causes including reduced muscle mass, denervation, or loss of coordination. CC, central connection; Nl, normal; PM, perineal membrane; PPM, puboperineal muscle; PRM, puborectal muscle; STP, superficial transverse perineal muscle; Ur, Urethra; Vag, vagina

The spatial relationships and connections for each element determine its mechanical action. Figure 4 displays a 3D model made from a nulliparous woman with normal support. It demonstrates the connections and relationships between the perineal membrane, vaginal wall, and medial pubococcygeal muscles. (The consequences of failure of any one element are shown in Fig. 5.) The second row of images show alterations in the perineal membrane that reflect changes in the perineal complex and are abstracted from previous work [15].

### Conceptual Diagrams

Figure 5 presents a simplified diagram showing different examples of failure sites and their combinations. The 2D illustration does not reflect the 3D consequences of these failures; however, it does provide a more concise shorthand for understanding how different combinations of failures can result in hiatal enlargement. The puboperineal portion of the pubococcygeal muscle is shown because it is the most medial portion related to hiatal closure. The puborectal muscle^1^ is included because it is spared with pubococcygeal muscle avulsion [16]. Therefore, it could still act to keep the hiatus closed when the perineal body is completely separated in a fourth-degree laceration independent of pubococcygeal injury. Given the number of different failure sites and the possibility of both partial and complete injuries, many different injury types can be present.

**Fig. 6:**
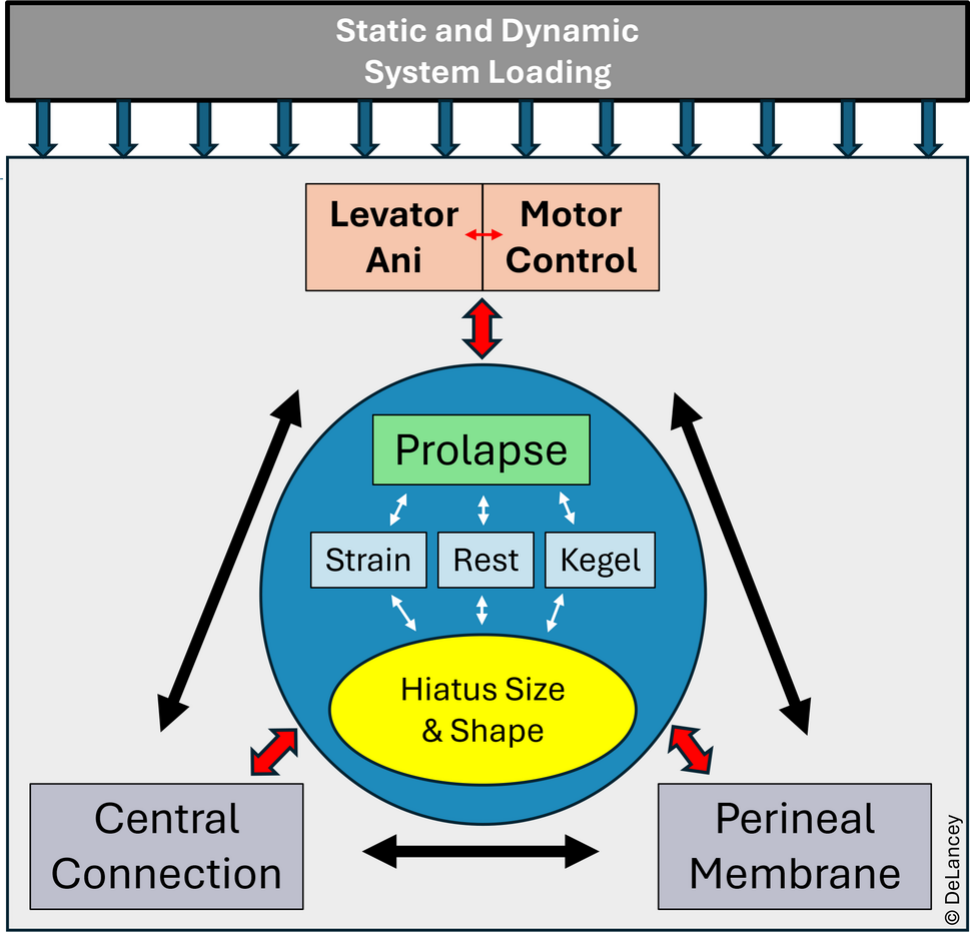
Factors associated with hiatal function. Schematic diagram of interactions among elements that affect hiatal size during system loading to show the many ways structures can interact with one another and affect hiatal size and shape; how they are influenced by the dilating effect of prolapse; and how they are affected by muscle activation state—rest, strain, and muscle contraction (“Kegel”)

Interactions among the structures involved in hiatal closure, as well as factors that affect hiatus size and shape and play a role in determining hiatal function, are presented in Fig. 6. The diagram organizes the different elements to provide a framework for understanding their interactions. Because motor control and levator ani muscle systems are so intimately related, they are combined in the model. There is, however, a great deal of complexity in the model and it is beyond the scope of this paper to fully explore all of the relationships. The outer three elements describe the key structures involved, with the loads placed upon them represented at the top of the diagram. The three elements are related to one another and interact as indicated by the bi-directional black arrows. Not only are there mechanical connections among these elements, but if one is damaged during vaginal birth, others are likely to be damaged as well. Within the blue circle are functional factors that all interact in determining measurements of hiatus size and shape at any given time. The loss of support in Level I and II that allows the vaginal walls to descend and exert a dilating effect on the hiatus is indicated by the “prolapse” element.

### Functional Aspects

Table 2 summarizes the functional anatomy, action, and mechanisms related to failure and the biomechanical consequences of injury to the three system components.

**Table 2:** Functional anatomy, action, mechanisms, and consequences of hiatal failure

Elements	Functional Anatomy	Action	Failure Mechanisms	Biomechanical Consequences
	Questions			
	*What is it?*	*What does it do?*	*How does it fail?*	*So what?*
Levator Ani Muscle (pubococcygeal muscle)	• Originates from pubis near superior pubic ramus • Attaches to the vagina, passes through the perineal body, and inserts into the anus	• Constricts the urogenital hiatus creating HPZ and levator hiatuses, drawing structures up and toward the pubic bone • Prevents Level I and II CT loading	• Birth-related avulsion • Weakening with age • Denervation • Suboptimal length for force generation	• Loss of AP closure force and with avulsion, widening of hiatus and loss of lifting vectors from attachments to vagina, perineum, and anus
Motor Control	• Afferent (sensory) and efferent (alpha motor) neurons • Fusimotor system (gamma motor neurons, intrafusal muscle fibers, muscle spindles) • Golgi tendon organs • Sacral reflex arcs • Cortical control (somatosensory and motor cortices)	• Sets active “resting tone” and modulates muscle activity during stressor events to keep hiatus closed during increased load	• Nerve injury reduces innervated muscle bulk • Complete or partial loss of automatic activation	• Reduced closure force • Lack of activation or insufficient activation during stressors connective tissue repetitive loading stretching/failure
Perineal Membrane, Endopelvic Fascia, and Central Connection	• PM origin from anterior pubic rami • Attaches to vaginal wall and levator ani bilaterally • Puboperineal part of LA passes through • Connects bilateral PM and superficial transverse perineal muscle • Rich in smooth muscle	• Prevents levator diastasis • Limits dorsal and caudal movement of perineal membranes with the attached vaginal wall, levator ani	• Birth-induced central rupture • Spreading over time with repetitive loading from prolapse • Alterations in material properties	• Diastasis of the levators and hiatal enlargement • Separation of perineal membranes loss of connections of BSM and STrP • Downward membrane rotation and hiatal widening

*AP* anterior–posterior *BSM* bulbospongiosus muscle, *CT* connective tissue *HPZ*, high pressure zone, *LA levator ani*, *PM*, perineal membrane; *StrP* superficial transverse perineal muscle

A framework that acknowledges the complexity of this system allows different elements to be isolated and assessed. For example, it is possible to produce a standard dilating force to stretch the hiatus to avoid the confounding effect of a prolapse and the variations between individuals in the strength of the Valsalva effort [17]. Likewise, measurements could be made in the operating room during muscle paralysis to establish properties in the absence of muscle activity. The UHCS model can guide individual research and allow the accumulation of data from several groups in the same way that the details of the hypothalamic-pituitary axis were worked out over decades. “There are increasing capabilities for studying network interactions in complex systems, and the UHCS model will be well-suited for these approaches—especially as we begin to accumulate more data” [18].

### Why Consider This a “System”

One of the benefits of system analysis is that it helps one understand how, when, and why a system with multiple elements fails. For example, many systems have redundancy to preclude a partial failure in any one element from resulting in total system malfunction (e.g., blockage of one coronary artery can be compensated by collateral circulation). A system may be “compensated” when non-critical failures exist in redundant elements, but “de-compensated” when a critical combination of element failures is reached. Moreover, there can be a “critical failure” in one essential component (e.g., a broken power line going into a house) as opposed to a failure in a non-essential component (e.g., a break in a wire supplying only one lamp). Engineering approaches, such as a criticality matrix, can be used to quantitatively assess the role of each system element to categorize 1) how critical each is to system function and 2) how frequently the elements fail. Because the UHCS is comprised of muscles, neuromotor control elements, and several fascial tissues, any one of them may sustain damage yet be compensated for by other structures (as illustrated in Table 1). An example is a chain of events when one part of the system fails, putting excessive strain on others. The intact structures may then compensate for a while, but when affected by age, they eventually fail. Had they not been carrying that greater-than-average load, they may never have failed. So, considering the UHCS as a system is useful because it allows one to study multiple element interactions objectively, often using dynamic statistical methods.

### Clinical Implications

At present, we do not know which patients need Level III perineal complex repair, which failures that need repair are present in each woman, which technique is most appropriate for each failure site and combinations of failures, or whether postpartum identification of Level III defects and early repair could help prevent pelvic organ prolapse. The use of posterior/perineoplasty repair among different surgeons in a Pelvic Floor Disorders Network trial ranged from 0% to over 90% during native tissue repair, and repair techniques vary widely [19, 20]. As the mechanisms of a disease are worked out and imaging and functional testing further developed, clinical trials of specific treatments can be developed to address a particular failure and determine the treatment’s success—such as how studies of obstetric anal sphincter injury diagnosis and repair flourished once endoanal ultrasound became available. That technique allowed investigators to assess what the repair needed to address and whether it was successful in restoring anatomy [21]. It is also likely that novel treatments and devices will need to be imagined and developed for problems that currently lack treatments. Table 3 lists some clinical considerations related to the distinct aspects of the UHCS.

**Table 3:** Clinical issues

Elements	Clinical Issues	Needs
Levator Ani Muscle (mainly pubococcygeal muscle)	• Preventing injury at birth • Muscle rehabilitation and strengthening • Develop and test novel interventions: → Surgical repair → Muscle replacement → Device development	• Identification criteria of at-risk women before labor and after delivery • Injury prevention trials • Identification of who will benefit from physical therapy • Innovation in surgery/device development
Neuromotor Control	• Prevention of nerve injury at birth • Motor skill training to replace automatic activation (Knack) • Neural excitability (spinal, cortical)	• Novel testing strategies to identify which part(s) of the neuromuscular control system is/are affected • Diagnostic criteria to identify affected individuals • Novel non-operative interventions (which interventions are most effective for specific defects) • Possible neuromodulation development
Perineal Membrane, Central Connection, and Other Endopelvic Fascia	• Prevention at birth • Potentially correctable surgically • Identification of failure feasible (3D ultrasound)	• Identify who might benefit from early repair • Evidence about successful anatomical restoration • Clinical trials comparing specific repair techniques with 3D ultrasound outcome assessment

Because most perineal complex damage occurs at the time of vaginal birth, it is an ideal environment for conducting clinical research into injury occurrence and prevention. In fact, a pilot clinical trial of a device to prevent avulsion of the levator ani muscle (the best-studied of the perineal complex structures) showed reduced levator injury [22]. Additionally, studies of perineal reconstruction with perineorrhaphy for a deficient perineum have shown at least symptomatic improvements in patients [23, 24]. Perineal complex structures could be studied clinically using 3D endovaginal ultrasound [25], which would permit identification of women who have been injured so that comparative effectiveness trials could guide practice.

To understand perineal complex failures and hiatal enlargement, we must recognize the need for further investigative work beyond relying on the POP-Q, which evaluates hiatus size only during straining. This work is needed, because the presence of a prolapse confounds these measurements, as shown in observations 3 and 4 (Table 1) where two women with equally large straining hiatus measures have quite different resting measures (1.5 and 6 cm). Measurements made at rest do show differences [26] between women with and without prolapse and should be considered in addition to those made during straining. Additionally, because it is hiatal area that is most biomechanically relevant, transverse diameter can easily be estimated in an analogous way during physical examination to assess cervical dilation [3] when imaging is not being performed.

While there is some early evidence that perineal reconstruction can lead to symptomatic improvement [23], more evidence is needed regarding what Level III defect is present and which specific repair techniques lead to symptomatic and biomechanical improvements.

### Research Implications

For any disease, research at the system, organ, tissue, cellular, molecular, and genetic levels is needed to permit innovation in both prevention and personalized diagnosis and treatment. It is beyond the scope of this article to review the Level III perineal complex research; however, a few observations can be made about research in different domains to highlight areas needing work.

There are several types of tissue that comprise the hiatal closure system. Classically, all tissues have normal characteristics that must be measured to understand their function. It is then possible to determine in whom they differ from normal. The pelvic floor is unique in many ways, so the assumption that tissue properties from other body regions can be used is incorrect. For example, the fact that most striated muscles have bony origins and insertions with an intervening joint that limits the length at which the muscle functions would not apply to the levator ani muscle, which does not have these limitations. Three-fold muscle lengthening during birth is unprecedented in the body.

Table 4 presents examples of research needs for tissues, biomechanical principles, and other factors. It is not intended to be exhaustive, but rather to illustrate the scope of research needed. The composition and properties of Level III connective tissues and the neural control systems are not as well studied as the pelvic floor muscles and are ideal areas for new discoveries. Because the connective tissues in Level III are unlike those in Levels I and II, the structures of the Level III perineal membrane and fascial tissues need to be evaluated. There is a considerable amount of smooth muscle within this tissue [27], raising the possibility of autonomic innervation that is yet unexplored. The remarkable changes that occur in these tissues in preparation for—and recovery from—vaginal birth are some of the most remarkable changes that occur in the human body, yet what triggers them and how they recover is unknown. While there was active interest in the neurobiology of pelvic floor disorders in the 1990 s, the study of the remarkable automatic responses of the pelvic floor in women with normal support remains much less developed.

**Table 4:** Research issues and current status^a^

Elements	Properties	Status
Levator Ani Muscle (pubococcygeal muscle)	• Muscle bulk/integrity/geometry • Resting tone and maximal force • Fatigability/response to loading • Length tension relationships • Histological abnormalities • Cellular and molecular mechanisms • Fiber type/sarcomere length • Injury threshold	+ + + + + + + + + + + + –-
Neural Controls	• Preprogrammed actions (“pre-activation” or “co-activation”) • Reflexes (spinal cord/brain) • Gamma motor system (length/tension control) • Nerve conduction velocity • Partial denervation-reinnervation	+ + + + + +
Connective Tissue: Perineal Membrane, Central Connection and Endopelvic Fascia	• Collagen type/elastin content/fiber organization • Stress/strain behavior (biaxial) • Tissue ripening before birth and reassembly postpartum • Response to repetitive loading (hypertrophy vs unraveling) • Injury threshold and location • Age-related changes • Tissue adaptation and triggering thresholds	± + –- –- –- –- –-
Biomechanics	• Injury failure site identification • Geometry and functional interaction among structures • Structural alignment/misalignment • System response to load and load distribution • Effect of individual and combinations of failures • Prolapse-hiatal interactions	+ + + + –- +
Other Factors	• Childbirth • Genetics affecting structure and response to damage/loading • Lifestyle demands • Comorbidities (diabetes, hypertension, connective tissue diseases, etc.)	+ + + + –- +

^a^Symbols indicate subjective assessment of the depth of current research from robust [+ + +] to absent [–-]

Our understanding of pelvic floor biomechanics has progressed, but most of that is related to Levels I and II. Hiatal closure has, to date, been considered as a single parameter. The biomechanics of how tissues work together in Level III are not currently known, so how a specific failure affects the system is unclear. For example, rupture of the central connection between the two sides of the perineal membrane can result in levator ani diastasis, placing the muscles in a suboptimal position that can make closure less efficient. This misalignment of structures limits each element’s ability to perform its function. As prolapse dilates the vagina, it elongates the levator ani muscles [9]. There is an optimal length for muscle contraction, and if it is exceeded, the generation of muscle force is reduced. That, in turn, results in more vaginal wall descent and greater elongation—and a vicious cycle ensues. Similarly, if the neural control system that stimulates muscle precisely at the optimal time for a load is broken—either because pre-activation is disabled or the afferent fibers lost—then muscles cannot provide protective support during loading.

The dynamics between damage to Level I and II resulting in vaginal wall descent and how that increased dilating pressure can open a normal hiatus needs to be considered. Conversely, an open hiatus allows excessive forces on Level I [28]and can cause descent. Increasing prolapse size over the decades of a woman’s life raises the questions of how forces, tissue properties, and tissue adaptation to increased loads interact to result in clinical prolapse. Disentangling these issues could be accomplished with creative study designs, but this research is yet to be done.

We have focused primarily on the perineal complex structures for this article. Of course, there are other key factors to consider, including how genetic factors affect each element of this system, how robust of a system is developed in an individual, and how these differences affect childbirth injury and the body’s ability to manage different lifestyles that alter the loads the pelvic floor must deal with. Systemic diseases that affect connective tissue, neural control, and muscle integrity will also need to be explored. Determining how these factors contribute to disease can benefit from an understanding of how the pelvic floor works. The mechanistic effect of tissue characteristics matter. For example, should connective tissues be compliant to avoid rupture or stiff to limit mobility? Only by gathering data on these issues will we be able to form a more complete understanding of hiatal failure—the most severe of the various failure sites.

### Concluding Remarks

When the specific failure sites and injury mechanisms that cause a symptom are determined, targeted treatment and novel interventions can be developed to specifically address the failure(s) present in each individual. These advances can lead to improved treatment outcomes and conserve resources for both patients and providers. The perineal complex has a multi-structural composition similar in complexity to the rotator cuff. Prolapse requires surgery three times more often than injury to the rotator cuff, but the science of rotator cuff injury is far more advanced than birth-related Level III injury. For instance, the search term “rotator cuff & pathophysiology” returns 2157 articles, and when surgery is required, specific injury sites are identified and then corrected during the operation. Research into the perineal complex and hiatal closure is just beginning, but it holds the possibility of yielding important discoveries that should advance prevention and treatment.

## Data Availability

Not relevant to this work.

## Footnotes

1 Some authors, especially those writing about ultrasound assessment of levator muscle damage, refer to the injured muscle as the “puborectal” muscle. The puborectal and pubococcyteal muscle are not the same according to *Terminologia Anatomica* and our research has shown that it is the pubococcygeal, not the puborectal, muscle that is damaged.
